# Research and application of medicines for treating liver fibrosis: current status and prospects

**DOI:** 10.3389/fphar.2025.1582258

**Published:** 2025-07-09

**Authors:** Shiqi Chen, Zhu Wu, Jing Zhang, Yuxin Lin, Jiaqi Xie, Dehui Yin, Ye Zhu

**Affiliations:** ^1^ Department of Traditional Chinese Medicine, The First Affiliated Hospital of Hainan Medical University, HaiKou, China; ^2^ College of Traditional Chinese Medicine, Hainan Medical University, HaiKou, China

**Keywords:** liver fibrosis, medicine therapy, biomedicine medicines, repurposed drugs, potential molecules, botanical drug metabolites, traditional Chinese medicine formulas, combined treatment

## Abstract

**Ethnopharmacological Relevance:**

Liver fibrosis is a common pathological consequence of multiple chronic liver diseases, making its pharmacological management a key area of medical research. Diverse classes of therapeutic agents offer distinct advantages and limitations. Notably, combination therapy has emerged as a prominent focus of contemporary investigation due to its potential to enhance treatment outcomes.

**Materials and Methods:**

As of 1 February 2025, a comprehensive literature search was conducted using PubMed and Web of Science, employing keywords related to liver fibrosis and its treatment. In accordance with ConPhyMP guidelines, one author assessed the quality of studies involving botanical drug metabolites.

**Results:**

This review synthesizes findings from 111 research articles, offering an overview of two primary classes of therapeutic agents and their integration with emerging technologies-namely mesenchymal stem cell-derived exosomes and nanoparticles. On one side, it discusses biomedicine-related therapies, including conventional biomedicine medicines, repurposed drugs, and investigational compounds. On the other, it addresses botanical-based treatments, encompassing traditional Chinese medicine (TCM) formulas and botanical drug metabolites. Both categories have shown promising therapeutic efficacy in clinical and preclinical settings.

**Conclusion:**

This review provides a comprehensive and detailed overview of pharmacological strategies for the treatment of liver fibrosis, shows the application and research status of different types of medicines, and provides a comprehensive perspective for current research directions. It points out the limitations of existing research and suggests that the clinical research of various medicines and combination therapies should be strengthened in the future, and the liver fibrosis model should be optimized to promote clinical transformation, which provides an important reference for future research directions.

## 1 Introduction

Liver fibrosis is a complex pathological response to chronic hepatic injury, characterized by the activation of hepatic stellate cells (HSCs) and excessive extracellular matrix (ECM) deposition, ultimately leading to fibrous tissue formation (Zhang et al., 2021). It represents the liver’s wound-healing mechanism in response to sustained damage and is a shared pathological endpoint across a spectrum of chronic liver diseases, including viral hepatitis and non-alcoholic fatty liver disease (NAFLD) (Henderson et al., 2020)—the latter now recognized as the most prevalent liver disease globally (Kuchay et al., 2020). The extent of hepatic fibrosis is a critical predictor of prognosis and mortality in chronic liver conditions, contributing significantly to global disease burden, healthcare costs, and the rising incidence of cirrhosis-related complications (O'Hara et al., 2020; Sanyal et al., 2023).

Despite its clinical importance, effective treatment of liver fibrosis remains challenging due to its multifactorial and dynamic pathogenesis, which involves complex signaling pathways and pro-fibrotic mediators such as transforming growth factor-beta (TGF-β) (Roehlen et al., 2020). Although several biomedicine pharmacological agents have been developed for liver fibrosis secondary to chronic liver diseases, their efficacy is often suboptimal, and adverse effects are common. Furthermore, many repurposed drugs that show promise in preclinical models have failed to yield meaningful outcomes in clinical trials (Gilgenkrantz et al., 2021). In contrast, certain Traditional Chinese Medicine (TCM) formulas have demonstrated definitive anti-fibrotic effects and are currently approved for clinical use in China (Li, 2020). Additionally, innovative drug delivery strategies—such as mesenchymal stem cell-derived exosomes (MSC-ex) and nanoparticles (NPs)—are being explored to improve hepatic targeting, enhance therapeutic efficacy, and reduce systemic toxicity (Feng et al., 2024; Liu J. et al., 2024).

**FIGURE 1:**
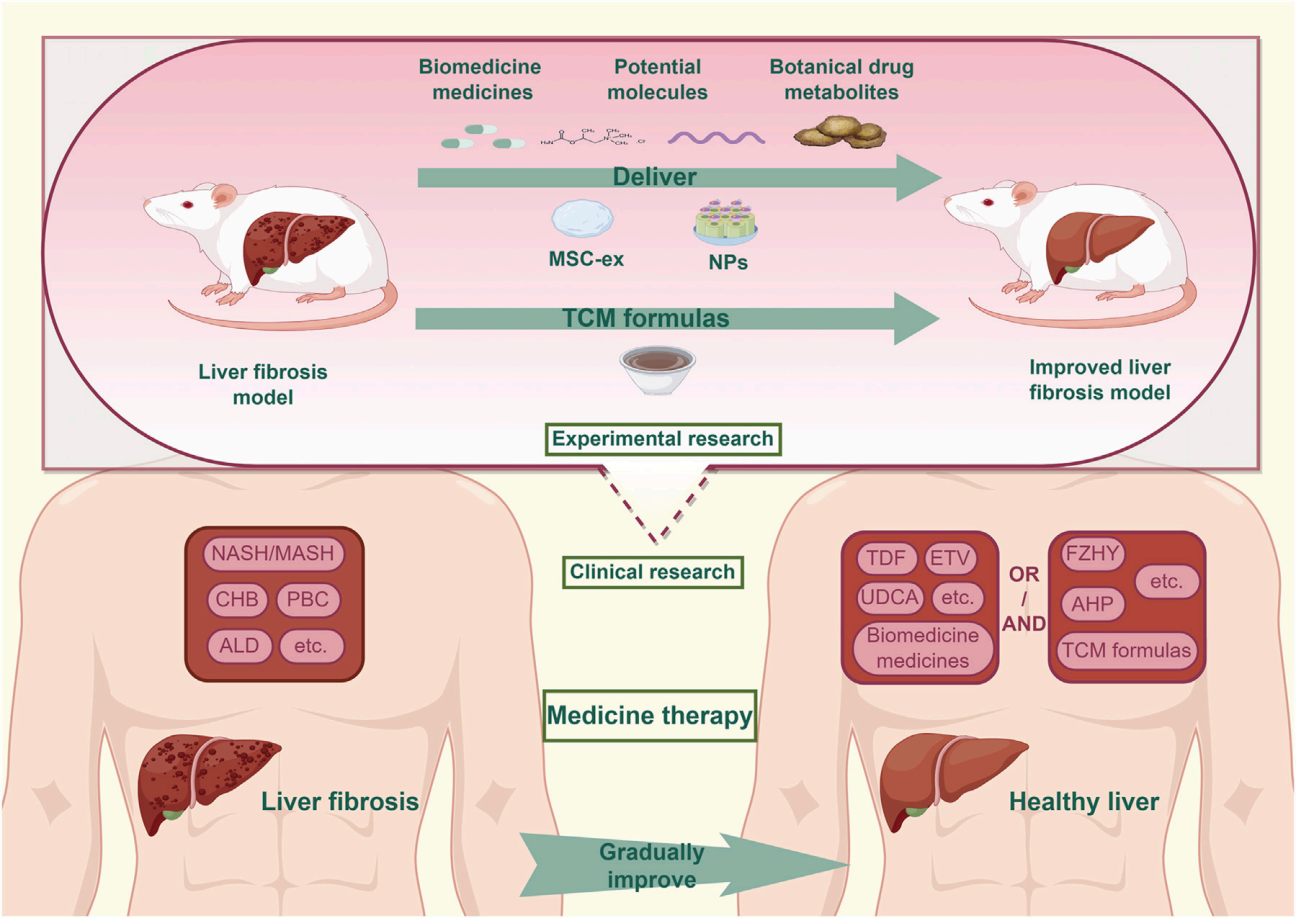
The situation of medicine treatment for liver fibrosis.In clinical practice, the combined treatment of Biomedicine medicines and TCM formulas has been confirmed as an effective strategy to address liver fibrosis caused by common chronic liver diseases. Currently, the field of experimental research are actively advancing the discovery of medicines for the treatment of liver fibrosis, focusing on areas such as repurposed drugs, potential molecules, botanical drug metabolites. MSC-ex and NPs technology provide strong support for translating Experimental research findings into clinical treatments. Abbreviation: NASH, Non-alcoholic Steatohepatitis; MASH, Metabolic Associated Fatty Liver Disease; CHB, Chronic Hepatitis B; PBC, Primary Biliary Cholangitis; ALD, Alcoholic Liver Disease; TDF, Tenofovir Disoproxil Fumarate; ETV:Entecavir; UDCA, Ursodeoxycholic Acid; FZHY, Fuzheng Huayu tablets; AHP, AnluoHuaxian pills; TCM, Traditional Chinese medicine.

This review presents a synthesis of recent advances in liver fibrosis treatment, drawing from studies published in the PubMed and Web of Science databases within the past 3 years. It systematically examines developments in biomedicine pharmacotherapy, drug repurposing, novel molecular candidates, botanical metabolites, and TCM-based interventions. These findings highlight the expanding landscape of anti-fibrotic strategies and the growing importance of integrative approaches. In particular, the convergence of MSC-ex and NP technologies with pharmacological agents represents a promising frontier in precision liver therapy. This paper aims to provide a comprehensive overview of current treatment modalities and to identify emerging directions for future research (Figure 1).

## 2 Methods

A systematic literature review was conducted as of 1 February 2025, encompassing both *in vitro* and *in vivo* experimental studies retrieved from PubMed and Web of Science. The search employed keywords such as liver fibrosis, treatment, and Traditional Chinese Medicine. Inclusion criteria were: (1) studies addressing the therapeutic effects or mechanisms of pharmacological agents in liver fibrosis using any combination of the specified keywords; (2) studies presenting original experimental data with clearly defined methodologies; and (3) full-text articles published in English. Exclusion criteria included duplicate records, irrelevant studies based on title and abstract screening, and publications lacking mechanistic insight or sufficient methodological detail. Based on these criteria, 102 studies were selected for analysis. Of these, 18 studies focusing on botanical drug metabolites were further evaluated for methodological quality using the ConPhyMP assessment tool. The evaluation was jointly performed by authors ZJ and LYX, with detailed results provided in the supplementary materials.

## 3 Pathological mechanisms of hepatic fibrosis

**FIGURE 2:**
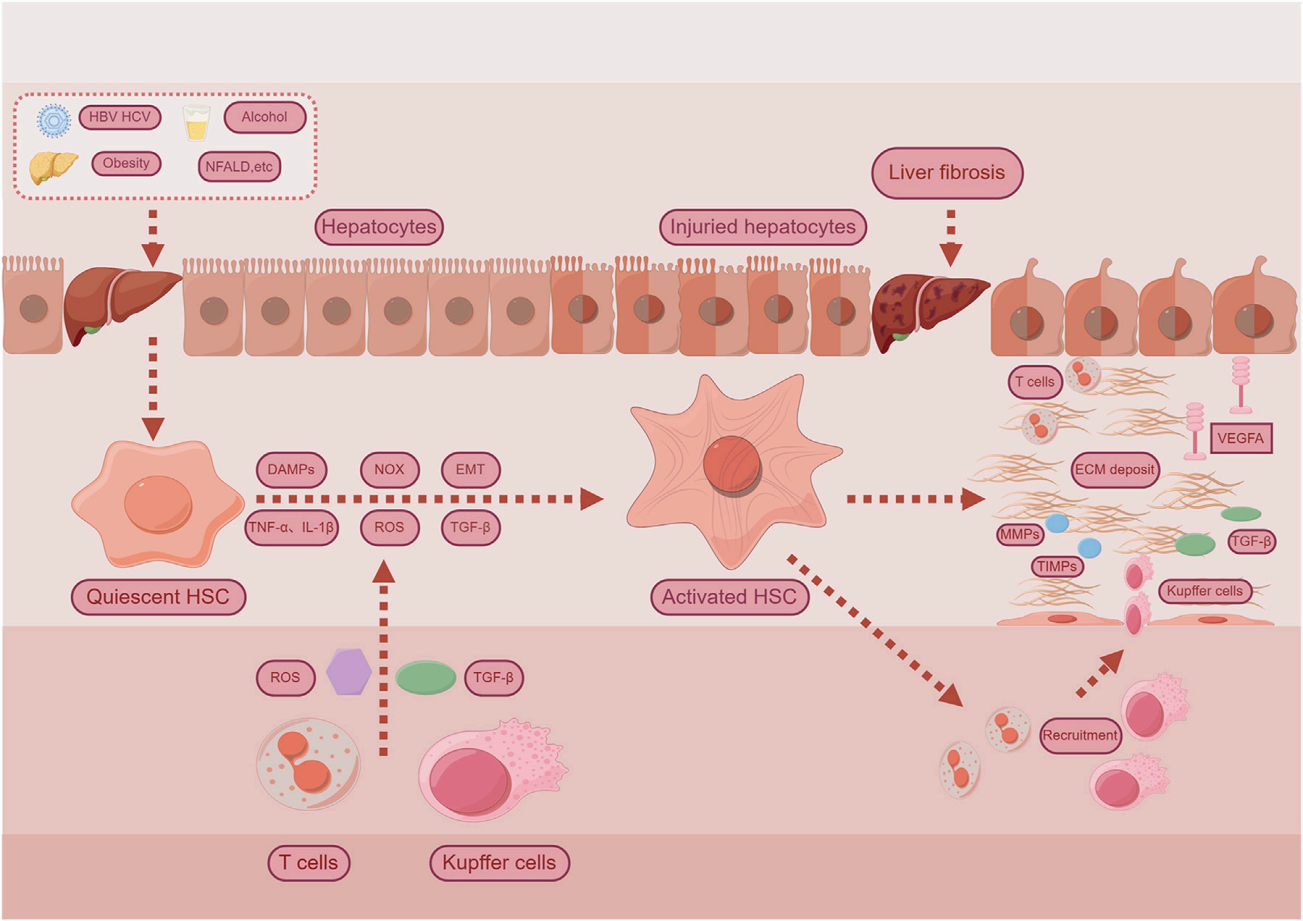
Examples for mechanisms for liver fibrosis. Chronic hepatocyte injury causes release of damage-associated patterns (DAMPs) and so on activate Hepatic stellate cells (HSCs) and recruit immune cells. Complex multidirectional interactions between activated HSCs and Kupffer cells, as well as innate immune cells promote trans-differentiation into proliferative and extracellular matrix (ECM) producing myofibroblasts. Abbreviations: TGF-β, Transforming Growth Factor Beta; NOX, Nicotinamide adenine dinucleotide phosphate-oxidase; TNF-α, Tumor Necrosis Factor-alpha; IL-1β, Interleukin-1-beta; ROS, Reactive Oxygen Species; EMT, Epithelial-Mesenchymal Transition; MMPs, matrix metalloproteinases; TIMPs, Tissue Inhibitors of Metalloproteinases; VEGFA, Vascular Endothelial Growth Factor A.

The progression of liver fibrosis is marked by the excessive accumulation of extracellular matrix (ECM) components (Iredale, 2007). Etiological factors such as toxins, metabolic disorders, or viral infections cause hepatocyte damage and immune cell infiltration, which, in turn, activate hepatic stellate cells (HSCs) and promote their differentiation into collagen-producing myofibroblasts (Zhang et al., 2016). Under physiological conditions, ECM synthesis and degradation remain in dynamic balance. However, in chronic liver disease, this balance is disrupted, favoring pro-fibrotic over anti-fibrotic signals. This shift induces myofibroblast proliferation, migration, contraction, and excessive ECM deposition. Moreover, vascular endothelial growth factor A (VEGFA) promotes pathological angiogenesis during fibrosis progression (Elpek, 2014; Shen H. et al., 2022). While producing ECM, activated HSCs also secrete matrix metalloproteinases (MMPs) and tissue inhibitors of metalloproteinases (TIMPs), the latter suppressing MMP activity and further reducing ECM degradation (Tacke and Zimmermann, 2014). In parallel, hepatocyte apoptosis and the release of damage-associated molecular patterns (DAMPs) directly stimulate HSC activation and initiate immune cell recruitment, including lymphocytes and macrophages. Kupffer cells, the liver-resident macrophages, can polarize into M1 or M2 phenotypes and secrete fibrogenic mediators such as transforming growth factor-beta 1 (TGF-β1) and reactive oxygen species (ROS). These mediators, along with ROS from neutrophils, enhance HSC activation and ECM synthesis (Ramachandran et al., 2012; Krenkel and Tacke, 2017). Oxidative stress further exacerbates fibrogenesis through NADPH oxidase (NOX)-mediated ROS production and the release of proinflammatory cytokines such as tumor necrosis factor-alpha (TNF-α) and interleukin-1 beta (IL-1β), which potentiate HSC activation and fibrotic remodeling (Ren et al., 2021). Additionally, epithelial-mesenchymal transition (EMT) contributes to the myofibroblast population (Chen et al., 2020). The TGF-β signaling pathway is pivotal in initiating and sustaining HSC activation and fibrogenesis (Dewidar et al., 2019). Beyond TGF-β, multiple signaling pathways have been implicated in modulating the fibrotic response. A comprehensive overview of the pathogenic mechanisms underlying hepatic fibrosis is illustrated in Figure 2.

## 4 Biomedicine treatment for liver fibrosis

### 4.1 Biomedicine medicines treatment for liver fibrosis

Clinically, liver fibrosis is commonly associated with non-alcoholic steatohepatitis (NASH), viral hepatitis, type 2 diabetes-related fatty liver disease, and primary biliary cholangitis (PBC). Standard pharmacotherapies—such as entecavir (ETV) for chronic hepatitis B (CHB) and ursodeoxycholic acid for PBC—have shown efficacy in mitigating fibrosis (Table 1). Nonetheless, the lack of fibrosis-specific targeted therapies continues to drive drug discovery efforts (Table 2).

**TABLE 1:** Clinical Studies related to the treatment of liver fibrosis by Western medicines.

Western medicines	Methods	Inclusion Criteria	Numbers	Dosing Schedule	Efficacy	Mechanism	References
Clinical trial used anti-liver fibrosis medicines							
Resmetirom	Clinical trial	Non-cirrhotic NASH adult patients with moderate to severe liver fibrosis	322	>100kg,oral 80mg; ≥100kg,oral 100mg once daily	Liver fibrosis improved for at least one stage, and most patients had mild adverse effects	Thyroid Hormone-beta agonist	Keam (2024)
Tirzepatide	Phase 2 clinical trial	Patients with MASH and F 2 or F 3(moderate or severe) fibrosis	190	Subcutaneous injection (5/10/ 15mg) or placebo once a week	Effectively improved the degree of liver fibrosis, common gastrointestinal adverse reactions	Glucose-dependent insulinotropic polypeptide and glucagon-like peptide-1 receptors	Loomba et al. (2024)
Pegozafermin	Phase 2 clinical trial	Patients with NASH and F 2 or F 3(moderate or severe) fibrosis	222	Subcutaneous injection of pegozamin(550 mg) twice daily or matched placebo	Effective in improving liver fibrosis but there were gastrointestinal adverse reactions	FGF21 analog	Loomba et al. (2023)
Survodutide	Phase 2 Randomized Trial	Patients with MASH and fibrosis stages F1-F3	293	Once-weekly subcutaneous injections of survodutide (2.4, 4.8, or 6.0 mg) or placebo.	Effective in improving liver fibrosis but there were gastrointestinal adverse reactions	Dual activation of glucagon receptor and GLP-1 receptor	Sanyal et al. (2024)
Rifaximin-α	Phase 2 clinical trial	Age 18-75 years, alcohol overdose for ≥1 year (≥24 g per day for women and ≥36 g per day for men) , biopsy-confirmed alcoholic liver disease, and no hepatic decompensation	136	Oral Rifaximin-α(550 mg) twice daily or matched placebo	It did not promote the regression of liver fibrosis, but slowed its progression	Repairing intestinal barrier and reduce inflammation	Israelsen et al. (2023)
Entecavir (ETV)/Tenofovir (TDF)	Clinical trial	Treatment-naïve CHB patients who had begun ETV or TDF	3277	Oral ETV/TDF	Although ETV users had higher initial liver fibrosis markers, ETV and TDF reduced liver fibrosis similarly over 6 months to 6 years	Antiviral	Chon et al. (2022)
Combined treatments							
Pioglitazone,exenatide,and metformin	6 year follow-up trial	Participants did not use medicines and met the American Diabetes Association standards for newly diagnosed type 2 diabetes (<2 years)	68	1000mg metformin, 5mg pioglitazone daily, and 5μg exenatide subcutaneously twice daily	Patients with type 2 diabetes receiving triple therapy had significantly lower fibrosis compared to those receiving standard treatment	Triple therapy (metformin/exenatide/pioglitazone	Lavynenko et al. (2022)
Hydronidone and entecavir	3-year, 2-phase randomized controlled trial	Patients with chronic hepatitis B and liver fibrosis	168	Either placebo or hydronidone (180,270, or 360 mg daily)	The combination of hydrodonitone and ETV, especially at a dose of 270 mg, significantly ameliorated CHB-related liver fibrosis	Hydronidone for Idiopathic pulmonary fibrosis	Cai et al. (2023)

**TABLE 2:** Experimental Studies related to the treatment of liver fibrosis by Western medicines.

Repurposed drugs	Methods	Key findings	References
Metformin	A review	Through cell and animal experiments, the therapeutic role of metformin in treating fatty liver disease and its associated liver fibrosis caused by type 2 diabetes has been demonstrated	Zhang et al. (2023a)
Pitavastatin	Wistar rats	Pit can effectively prevent liver fibrosis induced by TAA by reducing oxidative stress and exerting anti-inflammatory effects	Elbaset et al. (2024)
Montelukast	CCl4-induced mouse,LX-2 cell	Montelukast can reduce the indicators related to hepatic fibrosis by reducing the HSCs and inflammation	Pu et al. (2023)
Axitinib	CCl4-induced mouse,TGF-β1-induced HSCs	Axitinib can block the formation of specific proteins during the process of liver fibrosis	Li et al. (2023c)
Nimodipine	TAA-induced mouse	Nimodipine significantly alleviates liver fibrosis by remodeling the hepatic immune microenvironment	Guo et al. (2024)
Calcitriol	C57BL/6 mice	Calcitriol can alleviate liver fibrosis by acting on the newly discovered therapeutic target NS3TP1 through the TGF-β/Smad3 pathway	Shi et al. (2023)
Tetracycline	CCl4-induced mouse	Tetracycline inhibits the activation of HSCs by suppressing the phosphorylation process involving EphB1/2 and inhibiting the MAPK signaling pathway	Han et al. (2024)
Nitazoxanide	CCl4 and bile duct ligation-induced mice,LX-2 cells	Nitazoxanide can affect key signaling molecules in liver fibrosis, including AMPK, STAT3, and Smad2/3	Liu et al. (2024a)
Yohimbine	TAA-induced mouse,HLECs, LX2 cells, HepG2 cells	Yohimbine alleviates hepatic fibrosis by modulating oxidative stress and regulating the Wnt/β-catenin signaling pathway	Sharma et al. (2024)
Aspirin	Sprague-Dawley (SD) rats,HSC-T6 cells	Aspirin attenuates liver fibrosis by suppressing the TGF-β1/Smad signaling pathway	Sun et al. (2022)
Carfilzomib	CCl4-induced mouse,primary murine HSCs	Carfilzomib targets the NF-B/HIF-1αPathway to attenuate liver fibrosis	Fujiwara et al. (2024)
Ivermectin	Balb/c mice,CFSC cells	The anti-fibrotic effect of Ivermectin is mainly attributed to its ability to inactivate HSCs	Ying et al. (2022)
Dihydroergotamine	Molecular docking,LX-2 cells	Dihydroergotamine alleviates liver fibrosis through its action on the transforming growth factor β type II receptor	Zheng et al. (2023a)
Bemcentinib	C57BL/6J mice	Bemcentinib alleviates MASH-induced liver fibrosis by modulating the inflammatory response of Kupffer cells and CD8 T cells by blocking Axl	Grøndal et al. (2024)
Combined treatments			
Simvastatin and quercetin	Sprague-Dawley rats	Using quercetin together with simvastatin is more effective in treating liver fibrosis than their individual use, and the levels of sphk1 and nlrp3 mRNA decrease with the combination therapy	Salama et al. (2024)
MSC-ex and OCA	CCl4-induced mice	Exo-loaded OCA can enhance the protective effect of OCA on mice with liver fibrosis	Azizsoltani et al. (2023)
PM/HSCM@PLGA@Melatonin	C57BL/6 mice, AML12 cells	PM/HSCM@PLGA@Melatonin can enhance the bioavailability of Melatonin and boost its effectiveness in treating liver fibrosis	Bai et al. (2024a)
NPs and All-trans retinoic acid(ATRA)	CCl4 and methionine and choline deficient L-amino acid diet induced mouse	TM-ATRA/NPs induce both apoptosis and quiescence in activated HSCs and resulted in a significant improvement in fibrosis in mouse models	Xia et al. (2023)
(Hydroxychloroquine)HCQ@VA-Lip-Exo	BALB/c mice,HSCs T6,AML-12cells,BMSCs	HCQ@VA-LIP-EXO can reduce liver fibrosis by impacting autophagy	Zhang et al. (2023d)
HCQ@ROL-LNPs	TAA-induced mice,TGF-β-induced HSCs	HCQ@ROL-LNPs curb autophagy in activated HSCs by hydroxychloroquine(HCQ) and decrease the accumulation of ECM	Hou et al. (2024)
Sora@sirnp	Male C57BL/6 mice	Sora@siRNP has been shown to reduce liver fibrosis-related biomarker levels and improve drug bioavailability while reducing gastrointestinal side effects	Tran et al. (2022)
Silymarin-Chitosan nanoparticles	CCl4-induced rats	By embedding Sil into chitosan nanoparticles, these nanoparticles have shown more potent anti-fibrotic effects than chitosan nanoparticles alone	Abdullah et al. (2022)
HA@PRB/COL NPs	CCl4-induced mice	HA@PRB/COL NP can simultaneously disrupt the barrier formed by the deposition of collagen type I and specifically deliver probucol to inhibit the autophagic of HSCs, thereby alleviating liver fibrosis	Wang et al. (2024c)

#### 4.1.1 Clinical trial used anti-liver fibrosis biomedicine medicines

Resmetirom received initial regulatory approval in March 2024 for the treatment of metabolic dysfunction-associated steatohepatitis (MASH)/non-alcoholic steatohepatitis (NASH) and related fibrosis (Keam, 2024). In a phase 2 trial conducted by (Loomba et al., 2024), tirzepatide significantly improved MASH without exacerbating fibrosis after 52 weeks of treatment. In another study, (Loomba et al., 2023), evaluated pegozafermin, a fibroblast growth factor 21 (FGF21) analog, in NASH patients with moderate-to-severe fibrosis. After 24 weeks, fibrosis improvements were observed, although gastrointestinal side effects, notably nausea and diarrhea, were reported. (Sanyal et al., 2024). conducted a 48-week clinical trial assessing survodutide in patients with mild-to-moderate NASH-associated fibrosis. The drug demonstrated superiority over placebo in improving NASH features and preventing fibrosis progression, albeit with significant adverse effects. A follow-up study by (Lavynenko et al., 2022) focused on hepatic fibrosis in individuals with type 2 diabetes. In a large-scale study involving more than 3,000 patients with CHB and hepatic cirrhosis, (Chon et al., 2022), reported that ETV initially led to slight elevations in fibrosis biomarkers. However, long-term treatment (6 months–6 years) with ETV or tenofovir (TDF) yielded comparable efficacy in fibrosis reduction. The study also highlighted that combination therapy with metformin, exenatide, and pioglitazone was more effective in attenuating hepatic fibrosis than conventional stepwise regimens. Finally, in a 5-year randomized, double-blind, placebo-controlled phase 2 trial, (Israelsen et al., 2023), investigated pharmacologic interventions for patients with alcohol-related liver disease. Recent investigations suggest that Rifaximin-α may slow the progression of liver fibrosis, though this finding requires validation in forthcoming multicenter phase 3 clinical trials. At present, clinical studies have been involved in the treatment of liver fibrosis caused by several common liver diseases. However, many of these clinical trials are limited by small sample sizes, which compromises the reliability and generalizability of their findings.

#### 4.1.2 Repurposed drugs

(Zhang et al., 2023a) reviewed therapeutic advances related to metformin in treating fatty liver disease and liver fibrosis in patients with type 2 diabetes, emphasizing both clinical potential and underlying mechanisms. (Elbaset et al., 2024). demonstrated that pitavastatin possesses anti-fibrotic properties by attenuating oxidative stress and inflammatory signaling *via* modulation of the NF-κB and PI3K/AKT pathways. Similarly, (Pu et al., 2023), showed that montelukast effectively reduces fibrosis in murine models by inhibiting hepatic stellate cell (HSC) activation and inflammatory responses. (Li et al., 2023c). found that axitinib mitigates liver inflammation and fibrosis through dual mechanisms: suppression of HSC activation and enhancement of mitochondrial complexes I and III function. Further evidence supports the anti-fibrotic potential of other repurposed agents. (Guo et al., 2024). reported that nimodipine alleviates hepatic inflammation and fibrosis by modulating the liver’s immune microenvironment. (Shi et al., 2023). found that calcitriol regulates HSC activation, proliferation, and differentiation by downregulating NS3TP1 expression. (Han et al., 2024).showed that tetracycline inhibits activated HSCs by targeting EphB1/2 phosphorylation and the MAPK1/2 pathway. According to (Liu K. X. et al., 2024), nitazoxanide acts on key fibrotic signaling molecules including AMPK, STAT3, and Smad2/3, with follow-up studies confirming its efficacy in ameliorating fibrosis through these mechanisms. (Sharma et al., 2024). demonstrated that yohimbine reduces fibrosis and inflammation by modulating the JNK/Wnt/β-catenin pathway. Early evidence by (Jiang et al., 2016) indicated that aspirin decreases fibrosis indices in adult chronic liver disease patients in the US (Sun et al., 2022), a mechanism later confirmed in rat models *via* modulation of the TGF-β/Smad pathway. (Fujiwara et al., 2024). reported that carfilzomib reduces fibrotic marker expression in HSCs and attenuates CCl_4_-induced liver fibrosis. (Ying et al., 2022). identified ivermectin as an anti-fibrotic agent acting primarily through modulation of HSCs. (Zheng K. X. et al., 2023). employed molecular docking to identify dihydroergotamine as a potential TGF pathway modulator, with subsequent cellular assays confirming its anti-fibrotic effect.

Many of these agents, initially developed for unrelated conditions, demonstrate efficacy by modulating key pathogenic pathways involved in hepatic fibrosis. For example, the well-established links between diabetes, dyslipidemia, and liver fibrosis suggest that hypoglycemic and hypolipidemic drugs may exert secondary anti-fibrotic effects. However, it is important to recognize that commonly used experimental models, such as CCl_4_ and TAA-induced liver fibrosis, do not fully replicate the pathophysiological complexity of human liver fibrosis. This discrepancy may hinder the direct translation of preclinical findings into clinical practice, highlighting the need for more physiologically relevant models in fibrosis research.

#### 4.1.3 Combined treatments

In a 3-year, two-phase randomized controlled trial involving 168 patients, (Cai et al., 2023), demonstrated that Hydronidone combined with ETV significantly improved liver fibrosis associated with chronic hepatitis B, with 270 mg identified as the optimal dosage. (Salama et al., 2024). reported that combination therapy with Quercetin and Simvastatin was more effective than monotherapy in treating liver fibrosis, as evidenced by reduced expression of sphk1 and nlrp3 mRNA, indicating inhibition of the SphK1/NLRP3 signaling pathway. (Azizsoltani et al., 2023). explored mesenchymal stem cell-derived exosomes (MSC-ex) as a delivery platform for Obeticholic Acid (OCA), enhancing targeted delivery and mitigating side effects while effectively alleviating liver fibrosis in mouse models. Similarly, (Bai Y. et al., 2024), employed nanotechnology to improve Melatonin bioavailability, developing PM@PLGA and HSCM@PLGA nanoparticles that exhibited enhanced anti-fibrotic efficacy in animal models. (Xia et al., 2023). designed biomimetic nanoparticles (TM-ATRA/NPs) encapsulating all-trans retinoic acid (ATRA) within LX2 cell membrane-derived vesicles expressing TRAIL, which induced apoptosis and quiescence in activated hepatic stellate cells (HSCs), thereby ameliorating fibrosis in a mouse model. (Zhang Y. W. et al., 2023). developed HCQ@VA-Lip-Exo, a vitamin A-modified hybrid nanobiomimetic delivery system that effectively targeted activated HSCs, suppressed autophagy, and reduced extracellular matrix (ECM) synthesis and deposition. (Hou et al., 2024). further demonstrated that HCQ@ROL-LNPs selectively inhibited activated HSCs. (Tran et al., 2022). showed that sora@sirnp nanoparticles enhanced sorafenib bioavailability, producing anti-fibrotic effects while reducing gastrointestinal side effects. In another nanomedicine approach, a previous study identified silymarin-chitosan nanoparticles as a potent anti-fibrotic nanoformulation (Abdullah et al., 2022) that upregulated protective liver miRNAs and downregulated fibrosis-related markers such as TGFβR1, COL3A1, and TGFβR2. (Wang X. et al., 2024). developed HA@PRB/COL nanoparticles, a hyaluronic acid-based delivery system that targeted HSCs via CD44 receptors and facilitated ECM collagen I degradation through collagenases (COLs), effectively preventing HSC activation. Overall, combining conventional biomedicine medicines with MSC-ex or nanoparticles holds considerable promise for treating liver fibrosis by enhancing bioavailability, improving targeting, and reducing side effects. However, current studies often emphasize efficacy while overlooking adverse effects, limiting the comprehensiveness of research.

### 4.2 Potential molecules treatment for liver fibrosis

The evolving landscape of liver fibrosis research underscores the limitations of conventional biomedicine therapies in achieving cellular specificity. In response, there is growing interest in developing novel therapeutics based on protein and nucleic acid molecules (Table 3).

**TABLE 3:** Studies related to the treatment of liver fibrosis by Potential molecules.

Potential molecules	Methods	Key findings	References
Protein molecules			
S100A16	S100a16 knockdown and transgenic mice	Inhibition of the expression of CXCR4 to prevent liver fibrosis	Zhang et al. (2022b)
Igf2bp2	CCl4-induced mice	The knockdown of Igf2bp2 through the PI3K/Akt pathway targeted regulation of Tgfbr1 could improve liver fibrosis	Xu et al. (2022)
FGF18	CCl4-induced mice,LX-2 cells	FGF18 attenuates liver fibrosis through the SMO-LATS1-YAP signaling pathway	Tong et al. (2022)
PCSK9	CCl4-induced mice,AML12 cells	Anti-PCSK9 alleviates liver inflammation and fibrosis by regulating the AMPK/mTOR/ULK1 pathway and inhibiting hypoxia-induced hepatocyte autophagy	Ning et al. (2023)
Carnitine palmitoyltransferase 1A(CPT1A)	SiCPT1A mice,LX-2 cells	Silencing CPT1A reduces mitochondrial activity and prevents HSCs activation	Fondevila et al. (2022)
Mixed lineage kinase domain-like protein(MLKL)	Mlkl-knockout mice, LX-2 cells	Specific knockdown of Mlkl reduces hepatocyte necroptosis and HSCs activation	Guo et al. (2022)
SNS-032	C57BL6 mice,LX-2 cells	SNS-032 alleviates liver fibrosis by inhibiting cyclin-dependent kinase9	He et al. (2022)
GPR65	GPR65-KO mice,LX-2cells	Knockout GPR65 alleviates hepatic inflammation and fibrosis by suppressing the JNK and NF-κB pathways	Zhang et al. (2023c)
Sema4D	C57 BL/6J mice, LX-2 cells	Sema4D can inhibit the expression of AOX1 to resist liver fibrosis	Wang et al. (2023a)
GDF15	Gdf15 knockout mouse	GDF15 alleviates liver fibrosis through metabolic reprogramming of Macrophages to gain anti-inflammatory activity	Li et al. (2023d)
TM7SF3	TM7SF3KO mice,LX-2 cells	TM7SF3 is crucial for alleviating MASH-related liver fibrosis through the regulation of TEAD1 transcription factor activity	Isaac et al. (2024)
alectin 3-binding protein(lgals3bp)	C57BL/6J mice,Mouse HCC and human HCC cells	lgals3bp alleviates liver fibrosis by modulating the TGF-β signaling pathway	Kim et al. (2024)
IGNP-JQ1(The anti-fibrosis agent targeting growth factor 2 receptor)	CCl4 and Methionine choline deficiency diet-induced mice	The delivery of JQ1 by IGNP targets the activation of HSCs which helps alleviate liver fibrosis	Li et al. (2023a)
Nucleic acid molecules			
BMS-986263	A randomized, placebo-controlled phase 2 trial	BMS-986263 reduces the expression of hsp47 mRNA by delivering small interfering RNA, which helps improve the Metavir and Ishak scores of liver fibrosis	Lawitz et al. (2022)
MiR-190b-5p and miR-296-3p	Mouse models (toxin, cholestasis and MASH)	A notable decline in the expression of miR-190b-5p and miR-296-3p is observed in liver fibrosis	Markovic et al. (2024)
LncRNA H19-EZH2	Mdr2, bile duct ligation (BDL) and CCl4 mice	3-DZNeP specifically targeting the lncRNA H19-EZH2 thereby combating liver fibrosis	Li et al. (2023e)
Uridine	C57BL/6J mice,AML12 and NCTC1469 cells	Uridine can regulate the activity of liver-associated cells, thereby effectively reducing the accumulation of collagen in the liver and reducing α-SMA, type I collagen, and fibronectin levels	Zheng et al. (2022)
MSC-ex and miR-27b-3p	BALB/c female mice,LX-2 Cells	MSC-ex attenuates hepatic fibrosis by suppressing the YAP/LOXL2 signaling pathway by delivering miR-27b-3p	Cheng et al. (2023)
MSC-ex delivered miR-148a	C57BL/6 J mice,hUC-MSCs	MSC-ex delivers miR-148a to engage the KLF6/STAT3 signaling pathway to modulate intrahepatic macrophage function and thus exert mitigate hepatic fibrosis effects	Tian et al. (2022)
MSC-sEVs and miR-4465	CCl4-induced mouse models,LX-2 and HepG2 Cells	MSC-sEVs attenuate hepatic fibrosis by altering the delivery of miR-4465 into HSCs by altering LOXL2	Wang et al. (2024d)
Lipid nanoparticles (LNPs)	CCl4 induced mouse,LX-2 Cells	LNPs provide an effective therapeutic method for patients with liver fibrosis who are not suitable for protein injection therapy by delivering mRNA for therapeutic proteins	Shan et al. (2024)
AA-lipid-like lipid nanoparticles(A-LNP)	CCl4-induced mouse,3T3-GFP/H2.35 Cells	AA-LNP is more effective at attenuating liver fibrosis by targeting RNA delivery to activated fibroblasts than traditional LNPs	Han et al. (2023)
P138Y LNP and siGTSE1	C57BL/6 mice	P138Y LNP delivers siGTSE1 to hepatocytes and significantly reduces collagen accumulation, being more effective than conventional LNPs	Jeong et al. (2024)
SiRNA and Nanoparticles	HFCMCD and HFHC-induced mice,HSC-T6 cells	Using Nanoparticles to deliver siRNA to block the IL-1β/ERK signaling pathway can effectively inhibit the activated HSCs, leading to an alleviation of liver fibrosis	Zhang et al. (2023b)

#### 4.2.1 Protein molecules

(Zhang et al., 2022b) identified S100A16 as a suppressor of CXCR4 expression, which attenuated hepatic stellate cell activation by inhibiting the ERK1/2 and AKT signaling pathways. (Xu et al., 2022). demonstrated that silencing Igf2bp2 ameliorated fibrosis by modulating Tgfbr1 through the PI3K/Akt pathway. (Tong et al., 2022). revealed that FGF18 mitigates liver fibrosis *via* the SMO-LATS1-YAP signaling axis. Similarly, (Ning et al., 2023), showed that anti-PCSK9 antibodies reduce liver inflammation and fibrosis by modulating the AMPK/mTOR/ULK1 pathway and inhibiting hypoxia-induced hepatocyte autophagy. (Fondevila et al., 2022). reported that *CPT1A* silencing diminished mitochondrial activity, thereby suppressing HSC activation. (Guo et al., 2022). showed that knockdown of *Mlkl* specifically reduced hepatocyte necroptosis and HSC activation. (He et al., 2022). proposed that SNS-032, a cyclin-dependent kinase nine inhibitor, alleviates fibrosis by inducing apoptosis and suppressing activated HSCs. (Zhang K. et al., 2023). demonstrated that GPR65 knockout reduces hepatic inflammation and fibrosis through inhibition of the JNK and NF-κB pathways. Finally, (Wang L. et al., 2023), found that Sema4D knockdown downregulates AOX1 and RARA, thereby modulating T helper cell balance and counteracting liver fibrosis. (Li X. et al., 2023). demonstrated that GDF15 mitigates liver fibrosis progression by reprogramming macrophage metabolism to favor anti-inflammatory phenotypes. Similarly, (Isaac et al., 2024), highlighted the role of TM7SF3 in attenuating nonalcoholic steatohepatitis (NASH)-related liver fibrosis through regulation of the transcription factor TEAD1. (Grøndal et al., 2024). showed that Bemcentinib alleviates MASH-induced liver fibrosis by targeting AXL, thereby modulating the inflammatory responses of Kupffer cells and CD8^+^ T cells. In another study, (Kim et al., 2024), identified Galectin-3-binding protein as a potential biomarker for distinguishing stages of hepatic fibrosis and demonstrated its anti-fibrotic effects via modulation of the TGF-β1 signaling pathway. Additionally, (Li F. et al., 2023), investigated the effects of riociguat-pretreated IGNP-JQ1 on hepatic stellate cells (HSCs) in two murine models of liver fibrosis. Their findings revealed that this intervention significantly reduced hepatic fibrosis by enhancing substance exchange efficiency within liver tissue. Collectively, these studies have found that different molecular targets have intervening effects on liver fibrosis, but further exploration of their specific mechanisms of action is still needed.

#### 4.2.2 Nucleic acid molecules

Nucleic acids, particularly small interfering RNA (siRNA) and microRNA (miRNA), play pivotal roles in regulating liver fibrosis by modulating gene expression and signaling pathways. These molecules have emerged as promising therapeutic agents capable of halting fibrosis progression through targeted gene silencing. (Lawitz et al., 2022). administered BMS-986263—an siRNA targeting HSP47 mRNA—to 61 HCV-SVR patients with advanced liver fibrosis, reporting improvements in both METAVIR and Ishak scores. Infusion-related reactions were the most common adverse events. (Markovic et al., 2024). observed significant reductions in miR-190b-5p and miR-296-3p levels in murine and human liver fibrosis models, identifying hyaluronan synthase 2 (HAS2) and integrin alpha-6 as novel therapeutic targets. (Zhao et al., 2019). provided a comprehensive review of siRNA- and miRNA-based strategies for liver fibrosis, elucidating their mechanisms and therapeutic potential. Beyond canonical nucleic acids, researchers are also investigating related biological processes. (Li X. J. et al., 2023). showed that H19-regulated EZH2 reprograms H3K27me3, activating HSCs, promoting epithelial–mesenchymal transition (EMT), and triggering Wnt/β-catenin signaling—suggesting that disrupting this interaction could represent a novel therapeutic strategy. (Zheng et al., 2022). found that uridine modulates hepatic cell activity, reducing collagen accumulation and downregulating the expression of α-SMA, type I collagen, and fibronectin. (Cheng et al., 2023). reported that mesenchymal stem cell (MSC)-derived exosomes alleviate hepatic fibrosis by delivering miR-27b-3p to inhibit the YAP/LOXL2 signaling axis. Similarly, (Tian et al., 2022), showed that MSC-derived exosomes transporting miR-148a modulate intrahepatic macrophage activity via the KLF6/STAT3 pathway, thereby reducing fibrosis. (Wang Y. et al., 2024). further demonstrated that MSC-derived small extracellular vesicles modulate LOXL2 to facilitate miR-4465 delivery into HSCs, contributing to anti-fibrotic effects. Several nanoparticle-based delivery platforms have also shown promise. (Shan et al., 2024). used retinoid-derived lipid nanoparticles (LNPs) to deliver therapeutic protein mRNA in three NASH-associated liver fibrosis models, achieving enhanced protein retention and reduced systemic toxicity. (Han et al., 2023). developed AA-lipid-like LNPs that improved RNA delivery efficiency to activated fibroblasts and exhibited superior transmission compared to conventional LNPs. (Jeong et al., 2024). demonstrated that engineered LNPs delivering siGTSE1 to hepatocytes significantly reduced collagen accumulation. In a related study, (Zhang C. et al., 2023), observed IL-1β upregulation in two mature NASH mouse models and showed that siRNA-loaded nanoparticles targeting the IL-1β/ERK pathway effectively inhibited HSC activation and attenuated fibrosis. By targeting specific genes, nucleic acid molecules can regulate gene expression and signaling pathways, and achieve precise gene silencing. Not only do they improve symptoms, but they may also slow or even reverse the fibrosis process. However, the full mechanism of their role in disease progression needs to be further explored.

## 5 Botanical drug metabolites treatment for liver fibrosis

In medicinal research, clinical observations and empirical findings are fundamental to drug development. Botanical drugs has accumulated extensive clinical experience in managing liver fibrosis, offering a valuable foundation for pharmacological investigations. This body of knowledge suggests that specific botanical drug metabolites may hold therapeutic potential against hepatic fibrosis. Numerous reviews have explored phytotherapeutic strategies for hepatic fibrosis, often emphasizing key botanical drug metabolites such as polyphenols, phenolic acids, and flavonoids, along with their underlying mechanisms of action (Wenbo et al., 2024; Zhao B. et al., 2024). To advance this field, a phenotypic perspective on hepatic fibrosis—alongside a synthesis of recent mechanistic insights—may offer new avenues for therapeutic exploration. Table 4 presents a summary of studies investigating botanical drug metabolites in the treatment of hepatic fibrosis, with plant species cross-referenced against the MPNS database (https://powo.science.kew.org/).

**TABLE 4:** Studies related to the treatment of liver fibrosis by Botanical drug Metabolites.

Botanical drugs	Metabolites	Dose range/duration	Control group	Methods	Phenotype/Mechanism	References
[Campanulaceae;Lamiophlomis rotata]	polyphenolic glycosides	50-200mg/6 weeks	Olive oil(Negative control)	UPLC-Q/TOF/MS,CCl4-induced mice	Promoting the apoptosis of activated HSCs	Wan et al. (2023)
[Cucurbitaceae;Gynostemma pentaphyllum (Thunb.) Makino]	Gypenosides	25-50mg/4 weeks	Silymarin (Positive control) Olive oil(Negative control)	CCl4-induced mice,LX-2 Cells	Reducing ECM deposition in HSCs	Li et al. (2023b)
[Asphodelaceae;Aloe vera L.]	Aloin	20-40mg/8 weeks	Olive oil(Negative control)	C57BL/6 mice,HSC-T6 cells	Inhibiting the activation of HSCs	Bai et al. (2023)
[Ganodermataceae;Ganoderma lucidum]	Ganoderma lucidum Polysaccharide	200-480mg/4 weeks	Silymarin (Positive control) Olive oil(Negative control)	CCl4-induced mouse, TGF-β1-induced HSC-T6 cells	Influencing inflammation responses, cellular apoptosis, and ECM receptor activity	Chen et al. (2023)
	Triterpenoids	150-300mg/6 weeks	Silymarin(Positive control) Olive oil(Negative control)	UPLC-Q-TOF-MS,CCl4-induced mice,	Promoting the interaction between gut microbiota and metabolites	Zhang et al. (2024a)
[Apiaceae;Conioselinum anthriscoides 'Chuanxiong']	CXAL and CXPHL	10-18mg/1 weeks	Sham group(Negative control)	Network pharmacology,C57BL/6J mice,HIBE cells	Reducing collagen contraction force of HSCs	Li et al. (2024b)
[Araliaceae;Panax notoginseng (Burkill) F.H.Chen]	Notoginsenoside R1	25-100mg/6 weeks	Sham group(Negative control)	SD rats	Antioxidative and anti-inflammatory action	Gong et al. (2022)
[Gentianaceae;Gentiana scabra Bunge]	Gentiopicroside	40-80mg/1 weeks	MCC950(Positive control) Normal saline(Negative control)	C57BL/6 mice, RAW264.7 cells	Reducing hepatocyte pyroptosis	Yong et al. (2024)
[Paeoniaceae;Paeonia lactiflora Pall.]	Paeoniflorin	100-200mg/8 weeks	Lificiguat(Positive control) olive oil(Negative control)	Wistar rats,RAW264.7 cells	Affecting the polarization of macrophages	Liu et al. (2023b)
	Cryptotanshinone	40mg/5 weeks	Normal saline(Negative control)	CCl4-induced mice,LX-2 cells	Inhibiting fatty acid oxidation (FAO)	Li et al. (2024c)
[Fabaceae;Sophora flavescens Aiton]	Matrine	100mg/4 weeks	Normal saline(Negative control)	BALB/C mice, LX-2 and AML12 cells	Maintaining the levels of Heat Shock Protein 72 (HSP72), which is influenced by the modulation of the gut microbiota	Zhu et al. (2024)
[Celastraceae;Tripterygium wilfordii Hook.f.	Celastrol	0.25-1mg/3 weeks	Corn oil (Negative control)	ABPP, CETSA	Inducing ferroptosis in activated HSCs	Luo et al. (2022)
	Triptolide	50-100mg/8 weeks	GSK805(Positive control) Olive oil(Negative control)	CCl4-induced mice	Immunomodulation	Jiang et al. (2023b)
	Demethylzeylasteral	10-100mg/4 weeks	Corn oil (Negative control)	CCl4-induced mice,HSCs	Suppressing the proliferation, migration, and activation of HSCs	Chen et al. (2022)
[Fabaceae;Glycyrrhiza glabra L.]	Glabridin	10-40mg/8 weeks	Colchicine (Positive control) Corn oil (Negative control)	CCl4-induced mice	Activation of farnesoid X receptor;	Zhang et al. (2022a)
	Isoliquiritigenin	10-20mg/2 weeks	Pair feeding(Negative control)	C57BL/6 mice,HSC-T6 cells	Inhibition of ANXA2	Liu et al. (2023a)
[Lamiaceae;Origanum vulgare L.]	Didymin	0.5-2mg/6 weeks	4PBA(Positive control) Normal saline(Negative control)	Transcriptomics,CCl4-induced mouse	Alleviating Endoplasmic Reticulum Stress and Glycerophospholipid Metabolism	Li et al. (2022)

The combination of botanical drug Metabolites and other methods	Metabolites	Methods	Phenotype/Mechanism	References
[Zingiberaceae;Curcuma longa L.]	AgNPs and Curcumin	CCl4-induced liver fibrosis mouse model	The delivery of curcumin using green silver nanoparticles can effectively improve liver fibrosis	Elzoheiry et al. (2022)
[Apiaceae;Bupleurum marginatum Wall. ex DC.]	MnO2@PLGA/Ssb1 and Saikosaponin b1	Clinical specimens of cirrhosis patients and liver fibrotic mice,Balb/c mice	MnO2@PLGA/Ssb1 can enhance the utilization efficiency of SSB1 and, compared to the use of SSB1 alone, more effectively improve hypoxia-induced liver fibrosis	Dong et al. (2023b)
[Fabaceae;Sophora flavescens Aiton]	Mesoporous polydopamine nandrug(MPO) and Oxymatrine	C57BL/6 J mice,LX-2-cells	MPO encapsulation with oxymatrine can effectively eliminate excess ROS in liver tissue, balancing the TGF-β/Smads pathway, thereby alleviating liver fibrosis	Tang et al. (2023)
[Lamiaceae;Salvia miltiorrhiza Bunge ]	TSIIA-LNCs and Tanshinone IIA	Wistar rats	The modified LNC, when combined with TSIIA, can enhance its bioavailability and improve its anti-fibrotic effects	Ashour et al. (2021)
[Oleaceae;Forsythia suspensa (Thunb.) Vahl]	HA-mExo-FA and Forsythiaside A	Zebrafish larvae,LX-2-cells	HA-mExo-FA can overcome the limitations of pharmacokinetics and significantly improve liver fibrosis by inhibiting NLRP3-mediated pyroptosis	Gong et al. (2023)
[Paeoniaceae;Paeonia lactiflora Pall.]	Paeoniflorin and metformin	C57BL/6J mice	PAE and Met alleviate liver fibrosis by the TGF-β/Smad2 pathway	Meng et al. (2024)

### 5.1 Effects of botanical drug metabolites on the phenotype or mechanism of liver fibrosis

Aloin has demonstrated anti-fibrotic effects by inhibiting hepatic stellate cell (HSC) activation and attenuating CCL4-and TGF-β1-induced inflammatory responses in both *in vitro* and *in vivo* models (Bai et al., 2023). Activated HSCs contribute to fibrosis by promoting apoptosis, extracellular matrix (ECM) accumulation, and hepatocyte pyroptosis. (Wan et al., 2023). reported that the total polyphenolic glycosides of Lamiophlomis rotata suppress HSC proliferation *via* inhibition of the TGF-β/Smad pathway and enhance apoptosis of activated HSCs, thereby ameliorating liver fibrosis. Similarly, Gypenosides were shown to inhibit TGF-β-induced HSC activation and reduce ECM deposition, leading to fibrosis attenuation *in vivo* (Li et al., 2023b; Yong et al., 2024) found that Gentiopicroside (GPS), derived from Gentiana scabra Bunge, inhibits HSC activation by blocking the TLR4 and NLRP3 signaling pathways and suppressing hepatocyte pyroptosis. In a related study, (Luo et al., 2022), demonstrated that Celastrol alleviates hepatic fibrosis by inducing ferroptosis in activated HSCs through modulation of peroxiredoxins and heme oxygenase-1 (HO-1).

Emerging evidence suggests that botanical drug metabolites often target multiple fibrotic phenotypes simultaneously. For instance, (Chen et al., 2023), reported that Ganoderma lucidum polysaccharide (GLP) attenuates hepatic fibrosis by regulating inflammation, cell cycle progression, apoptosis, and ECM-receptor interactions. In another study, (Chen et al., 2022), showed that demethylzeylasteral inhibits HSC proliferation, migration, and activation, and downregulates fibrogenic gene expression by suppressing FAK and AKT phosphorylation through inhibition of the AGAP2-mediated signaling pathway.

Botanical metabolites also exert anti-fibrotic effects by reducing oxidative stress and inflammation driven by activated HSCs. Notoginsenoside R1, a metabolite of Panax notoginseng, alleviates fibrosis by suppressing HSC activation and downregulating NF-κB and MAPK signaling pathways, thereby exerting both antioxidant and anti-inflammatory effects (Gong et al., 2022). Some studies have extended beyond HSC-focused mechanisms. For example, CXAL and CXPHL, metabolites of Chuanxiong Rhizoma, mitigate hepatic fibrosis by modulating the CTCF–c-MYC–H19 pathway and reducing HSC-mediated collagen contraction (Li Y. et al., 2024). Additionally, (Liu Y. et al., 2023), demonstrated that Paeoniflorin exerts anti-fibrotic effects by modulating macrophage polarization *via* the NF-κB/HIF-1α signaling pathway.

This study also revealed novel mechanisms involved in the pathogenesis and mitigation of liver fibrosis. Triterpenoids have been shown to attenuate hepatic fibrosis by modulating NF-κB and TGF-β1/Smads signaling pathways, as well as by increasing the abundance of the gut microbiota genus Ruminococcus (Zhang J. et al., 2024). Similarly, Kuhuang alleviates liver fibrosis by influencing the gut microbiota’s regulation of hepatic interferon signaling and bile acid metabolism (Shen B. et al., 2022; Li Z. et al., 2024) reported that Cryptotanshinone (CTS) was reported to reduce liver fibrosis through the inhibition of p-STAT3/CPT1A-mediated fatty acid oxidation. (Jiang S. et al., 2023). showed that Triptolide exerts its anti-fibrotic effects by modulating T-helper (Th) and CD4^+^ T cell differentiation. According to (Li et al., 2022), Didymin, a metabolite derived from Origanum vulgare L., primarily mitigates hepatic fibrosis by reducing endoplasmic reticulum stress and altering glycerophospholipid metabolism. In addition to small-molecule metabolites, several protein-related mechanisms have emerged as promising anti-fibrotic targets. Glabridin alleviates inflammation and oxidative stress via PPARγ activation, thereby suppressing fibrotic progression (Zhang L. et al., 2022; Zhang L. et al., 2024) demonstrated that *Astragalus* saponins, through activation of the FXR receptor, downregulate key proteins implicated in hepatic fibrosis, particularly in cholestatic liver disease models. Isoliquiritigenin (ISL) inhibits ANXA2 expression and blocks the sphks/S1P/IL-17 signaling axis while suppressing STAT3 phosphorylation, leading to reduced α-SMA expression and fibrosis reversal (Liu N. et al., 2023). The investigation of botanical drug metabolites has expanded rapidly in recent years (Dan et al., 2024). However, focusing solely on mechanistic studies is insufficient to address clinical demands. If botanical drug metabolites that can act on the initial causes of primary liver diseases and effectively target liver fibrosis are successfully developed, for example, identifying botanical drug metabolites capable of simultaneously ameliorating cholestasis and liver fibrosis would substantially enhance research significance and translational potential. Mirroring the successful development trajectories of artemisinin and tanshinone, such efforts are critical to advancing liver fibrosis treatment. In contrast, existing agents such as colchicine, lificigat, and 4PBA lack fibrosis-specific efficacy, have limited clinical validation, and show weak mechanistic alignment, rendering them suboptimal as positive control drugs in experimental models.

### 5.2 Combined application

Combination strategies have demonstrated enhanced therapeutic efficacy. (Elzoheiry et al., 2022). addressed curcumin’s poor bioavailability using green silver nanoparticles (AgNPs) for targeted delivery in a mouse model, which resulted in significantly reduced liver fibrosis severity. (Dong H. et al., 2023). developed a multifunctional nanosystem (MnO_2_@PLGA/Ssb1), which integrates MnO_2_ with saikosaponin b1 (Ssb1) to augment its anti-fibrotic activity by scavenging excess H_2_O_2_ and relieving hypoxic stress. (Tang et al., 2023). utilized mesoporous polydopamine (MPO) for targeted drug delivery in both animal and cell models. MPO effectively reduced hepatic reactive oxygen species (ROS) and modulated the TGF-β/Smad signaling pathway, thereby attenuating fibrosis. Encapsulation of oxymatrine in MPO further enhanced therapeutic efficacy by restoring TGF-β/Smad signaling balance and reducing fibrotic progression. Similarly, (Ashour et al., 2021), demonstrated that lipid nanocapsules encapsulating Tanshinone IIA (TSIIA-LNCs) exerted superior anti-fibrotic effects compared to the free compound. (Gong et al., 2023). employed milk-derived exosomes modified with hyaluronic acid (HA-mExo-FA) to deliver Forsythiaside A. This nanocomplex targeted CD44 receptors on activated hepatic stellate cells, significantly inhibiting TGF-β1-induced LX2 cell proliferation, downregulating α-SMA expression, and promoting apoptosis (Meng et al., 2024). We found that many studies have been carried out on the combined application of mesenchymal stem cell exosomes (MSC-ex) or nanoparticle-based systems with botanical drug metabolites, potential molecules, and biomedicine medicines, however, there is still a lack of systematic comparative studies on the therapeutic effects of MSC-ex, nanoparticle-based systems and these medicines in the treatment of liver fibrosis. In the future, it is of great significance to carry out relevant comparative research.

## 6 Traditional Chinese medicine formulas treatment for liver fibrosis

Liver fibrosis, as a chronic progressive condition, has long been managed using TCM formulas, many of which are clinically validated. Increasingly, clinical studies are evaluating the integrative potential of TCM and biomedicine medicines in treating liver fibrosis. The TCM literature extensively documents the therapeutic efficacy of classical and empirically derived formulas. Recent rigorous investigations have elucidated the molecular mechanisms underlying these TCM-based interventions (see Table 5), further validating their relevance in modern hepatology.

**TABLE 5:** Studies exploring the therapeutic effects of TCM formulas on liver fibrosis.

TCM formulas	Ingredients	Methods	Key findings	References
TCM formulas mentioned in the “Guidelines”				
Fuzheng Huayu tablets(FZHY) /and Entecavir(ETV)	Salvia miltiorrhiza Bge. [Lamiaceae; Salviae miltiorrhizae radix et rhizoma], Panax notoginseng (Burk.) F.H.Chen [Araliaceae; Panacis notoginseng radix], Amygdalus persica (L.) Batsch [Rosaceae; Amygdali persicae semen], Carthamus tinctorius L. [Asteraceae; Carthami flos], Alism plantago-aquatica L. [Alismataceae; Alismatis rhizoma], Paeonia lactiflora Pall. [Paeoniaceae; Paeoniae radix rubra], Poria cocos (Schw.) Wolf [Polyporaceae; Poria], and Glycyrrhiza uralensis Fisch. [Fabaceae; Glycyrrhizae radix et rhizoma]	Single arm clinical objective performance criteria trial	After 48 weeks of combined treatment with FZHY and ETV, significant improvement in hepatic fibrosis was observed in 251 patients with advanced hepatic fibrosis due to CHB	Zhao et al. (2022)
		Review study	FZHY can be widely used in the treatment of liver fibrosis caused by various chronic liver diseases	Zhou et al. (2024)
		Clinical trial	Liver Qi Stagnation and Spleen Deficiency Syndrome are the most prevalent in patients by FZHY treatment for liver fibrosis	Dai et al. (2023)
Anluohua Xianwan (ALHX) /and ETV	Ophiocordyceps sinensis (Berk.) G.H.Sung, J.M.Sung, Hywel-Jones & Spatafora [Ophiocordycipitaceae; Ophiocordyceps sinensis corpus cum larva], Astragalus membranaceus (Fisch.) Bunge [Fabaceae; Astragali radix], Salvia miltiorrhiza Bunge [Lamiaceae; Salviae miltiorrhizae radix et rhizoma], Angelica sinensis (Oliv.) Diels [Apiaceae; Angelicae sinensis radix], Wolfiporia extensa (Peck) Ginns [Polyporaceae; Wolfiporiae sclerotium], Paeonia lactiflora Pall. [Paeoniaceae; Paeoniae radix alba], Prunus persica (L.) Batsch [Rosaceae; Persicae semen], Carthamus tinctorius L. [Asteraceae; Carthami flos], Pheretima aspergillum (E. Perrier) [Megascolecidae; Pheretima corpus siccum], and Glycyrrhiza uralensis Fisch. [Fabaceae; Glycyrrhizae radix et rhizoma]	Clinical trial	The combination therapy led to a significantly higher rate of liver fibrosis regression, as opposed to treatment with ETV alone	Liu et al. (2023c)
		A double-blind, randomized, placebo-controlled trial	Improvement in liver fibrosis was noted in 270 patients with early-stage disease after 48 weeks of treatment	Xiao et al. (2022)
Biejia-Ruangan (BR) and ETV	Trionyx sinensis Wiegmann [Trionychidae; Trionyx carapax], Belamcanda chinensis (L.) DC. [Iridaceae; Belamcandae rhizoma], Scutellaria baicalensis Georgi [Lamiaceae; Scutellariae radix], Bupleurum chinense DC. [Apiaceae; Bupleuri radix], Armadillidium vulgare Latreille [Armadillidiidae; Armadillidium corpus], Zingiber officinale Roscoe [Zingiberaceae; Zingiberis rhizoma], Rheum palmatum L. [Polygonaceae; Rhei radix et rhizoma], Paeonia lactiflora Pall. [Paeoniaceae; Paeoniae radix], Cinnamomum cassia Presl [Lauraceae; Cinnamomi ramulus], Descurainia sophia (L.) Webb ex Prantl [Brassicaceae; Descurainiae semen], Pyrrosia lingua (Thunb.) Farw. [Polypodiaceae; Pyrrosiae folium], Magnolia officinalis Rehder & E.H.Wilson [Magnoliaceae; Magnoliae cortex], Moutan cortex (Paeonia suffruticosa Andrews) [Paeoniaceae; Moutan cortex], Dianthus superbus L. [Caryophyllaceae; Dianthi herba], Campsis grandiflora (Thunb.) K.Schum. [Bignoniaceae; Campsis flos], Pinellia ternata (Thunb.) Makino [Araceae; Pinelliae rhizoma], Panax ginseng C.A.Mey. [Araliaceae; Ginseng radix], Eupolyphaga sinensis Walker [Corydiidae; Eupolyphaga corpus], Nidus Vespae [Vespidae; Nidus Vespae], Potassium Nitrate [Nitratrum; Nitrum], Catharsius molossus Linnaeus [Scarabaeidae; Catharsius corpus], and Prunus persica (L.) Batsch [Rosaceae; Persicae semen]	Clinical trial	500 patients with CHB and advanced hepatic fibrosis received a combination treatment of ETV and BR for seventy-two weeks, resulting in significant improvement in liver fibrosis	Rong et al. (2022)
Ganshuang granules	Bupleurum chinense DC. [Apiaceae; Bupleuri radix], Paeonia lactiflora Pall. [Paeoniaceae; Paeoniae radix alba], Citrus aurantium L. [Rutaceae; Aurantii fructus], Salvia miltiorrhiza Bunge [Lamiaceae; Salviae miltiorrhizae radix et rhizoma], Astragalus membranaceus (Fisch.) Bunge [Fabaceae; Astragali radix], Artemisia scoparia Waldst. & Kit. [Asteraceae; Scopariae herba], Wolfiporia extensa (Peck) Ginns [Polyporaceae; Wolfiporiae sclerotium], Atractylodes macrocephala Koidz. [Asteraceae; Atractylodis macrocephalae rhizoma], Glycyrrhiza uralensis Fisch. [Fabaceae; Glycyrrhizae radix et rhizoma], Curcuma longa L. [Zingiberaceae; Curcumae rhizoma], Isatis indigotica Fort. [Brassicaceae; Isatidis radix], and Gardenia jasminoides J.Ellis [Rubiaceae; Gardeniae fructus]	Sprague-Dawley (SD) rats,HSC-T6 cells	Experimental data indicate that Gan Shuang granules can reduce markers of liver fibrosis in rats, and its component naringenin may reduce fibrosis via the TGF-β/Smad signaling pathway	Wang et al. (2024a)
Other TCM formulas and Western medicines				
Ruangan granule(RG) and ETV	Trionyx sinensis Wiegmann [Trionychidae; Trionyx carapax], Panax notoginseng (Burk.) F.H.Chen [Araliaceae; Panacis notoginseng radix et rhizoma], Salvia miltiorrhiza Bunge [Lamiaceae; Salviae miltiorrhizae radix et rhizoma], Prunus persica (L.) Batsch [Rosaceae; Persicae semen], Astragalus membranaceus (Fisch.) Bunge [Fabaceae; Astragali radix], Atractylodes macrocephala Koidz. [Asteraceae; Atractylodis macrocephalae rhizoma], Wolfiporia extensa (Peck) Ginns [Polyporaceae; Wolfiporiae sclerotium], Curcuma aromatica Salisb. [Zingiberaceae; Curcumae radix], Citrus aurantium L. [Rutaceae; Aurantii fructus], Lobelia chinensis Lour. [Campanulaceae; Lobeliae herba], Schisandra chinensis (Turcz.) Baill. [Schisandraceae; Schisandrae fructus], and Phyllanthus urinaria L. [Phyllanthaceae; Phyllanthi herba]	A multicenter, randomized clinical trial	Among 240 patients with advanced liver fibrosis caused by CHB, a significant decrease in the extent of liver fibrosis was observed after 48 weeks of combined treatment with entecavir (ETV) and RG	Xing et al. (2023)
Bushen Huayu Decoction and ETV	Rehmannia glutinosa (Gaertn.) DC. [Orobanchaceae; Rehmanniae radix praeparata], Dioscorea opposita Thunb. [Dioscoreaceae; Dioscoreae rhizoma], Trionyx sinensis Wiegmann [Trionychidae; Trionyx carapax (processus cum aceto)], Cornus officinalis Siebold & Zucc. [Cornaceae; Corni fructus], Paeonia × suffruticosa Andrews [Paeoniaceae; Moutan cortex], Wolfiporia extensa (Peck) Ginns [Polyporaceae; Wolfiporiae sclerotium], Alisma plantago-aquatica L. [Alismataceae; Alismatis rhizoma], Aconitum carmichaelii Debeaux [Ranunculaceae; Aconiti radix lateralis praeparata], Cinnamomum cassia (L.) J.Presl [Lauraceae; Cinnamomi cortex], Achyranthes bidentata Blume [Amaranthaceae; Achyranthis radix], Plantago asiatica L. [Plantaginaceae; Plantaginis semen], and Panax notoginseng (Burk.) F.H.Chen [Araliaceae; Panacis notoginseng radix et rhizoma pulvis]	A retrospective study	The combination of Bushen Huayu Decoction and Entecavir is more helpful in improving liver fibrosis than Entecavir alone	Yu and Ge (2024)
Commercial Chinese polyherbal preparation(CCPP) and ursodeoxycholic acid(UDCA)		A comprehensive review and meta-analysis	The efficacy of treating PBC-related liver fibrosis: CCPP and UDCA > CCPP / UDCA	Bi et al. (2023)
Classic TCM formulas				
Dahuang Zhechong pills	Rheum palmatum L. [Polygonaceae; Rhei radix et rhizoma], Eupolyphaga sinensis Walker [Corydiidae; Eupolyphaga corpus], Prunus persica (L.) Batsch [Rosaceae; Persicae semen], Tabanus bivittatus Matsumura [Tabanidae; Tabanus corpus], Hirudo nipponica Whitman [Hirudinidae; Hirudo corpus], Holotrichia diomphalia Bates [Scarabaeidae; Holotrichia larva], Toxicodendron vernicifluum (Stokes) F.A.Barkley [Anacardiaceae; Toxicodendri resina], Scutellaria baicalensis Georgi [Lamiaceae; Scutellariae radix], Glycyrrhiza uralensis Fisch. [Fabaceae; Glycyrrhizae radix et rhizoma], Paeonia lactiflora Pall. [Paeoniaceae; Paeoniae radix rubra], Rehmannia glutinosa (Gaertn.) DC. [Orobanchaceae; Rehmanniae radix], and Prunus armeniaca L. [Rosaceae; Armeniacae semen]	Sprague-Dawley rats	Modulation of intestinal microbiota and their metabolites	He et al. (2024)
Jiawei Taohe Chengqi Decoction	Amygdalus persica (L.) Batsch [Rosaceae; Amygdali persicae semen], Cinnamomum cassia Presl [Lauraceae; Cinnamomi cassiae ramulus], Rheum palmatum L. [Polygonaceae; Rhei radix et rhizoma], Natrium sulfatum [Sulfatophora; Natrii sulfatis crystallum], and Glycyrrhiza uralensis Fisch. [Fabaceae; Glycyrrhizae radix et rhizoma]	C57/BL6 mice,LX-2 cells	Regulating Src/ERK/Smad3 signal pathway	Huang et al. (2023)
		CCl4-induced mice HF model,LX-2 cells	Mitigating liver fibrosis by suppressing HSCs activation through the TGF-β1/CUGBP1 and IFN-γ/Smad7 pathways	Ye et al. (2024)
		CCl4-induced mice, THP-1 cells and LX-2 cells	Suppressing the Notch signaling pathway, influencing macrophage reprogramming to prevent HSCs activation	Shao et al. (2024)
Guizhifuling pill	Cinnamomum cassia (L.) J.Presl [Lauraceae; Cinnamomi ramulus], Wolfiporia extensa (Peck) Ginns [Polyporaceae; Wolfiporiae sclerotium], Paeonia suffruticosa Andrews [Paeoniaceae; Moutan cortex], Prunus persica (L.) Batsch [Rosaceae; Persicae semen], and Paeonia lactiflora Pall. [Paeoniaceae; Paeoniae radix]	Male ICR mice	Modulating the Nrf2/HO-1 antioxidant system and inhibiting the NF-κB inflammatory	Baogui et al. (2022)
		CCl4-induced mice,LX-2 cells	Modulating the PTEN/AKT/mTOR signaling pathway	Yao et al. (2024)
Tao Hong Si Wu Tang	Salvia miltiorrhiza Bge. [Lamiaceae; Salviae miltiorrhizae radix et rhizoma], Carthamus tinctorius L. [Asteraceae; Carthami flos], Angelica sinensis (Oliv.) Diels [Apiaceae; Angelicae sinensis radix], Paeonia lactiflora Pall. [Paeoniaceae; Paeoniae radix alba], Rehmannia glutinosa (Gaertn.) Libosch. ex DC. [Orobanchaceae; Rehmanniae radix praeparata], and Ligusticum chuanxiong Hort. [Apiaceae; Chuanxiong rhizoma]	TAA-induced mice	Reversing ACSL4-mediated lipid accumulation and promoting mitophagy	Wu et al. (2024)
Si-Wu-Tang	Rehmannia glutinosa (Gaertn.) Libosch. ex DC. [Orobanchaceae; Rehmanniae radix], Paeonia lactiflora Pall. [Paeoniaceae; Paeoniae radix alba], Angelica sinensis (Oliv.) Diels [Apiaceae; Angelicae sinensis radix], and Ligusticum chuanxiong Hort.[Apiaceae; Chuanxiong rhizoma]	C57BL/6 J mice	Regulating lncRNA H19-dependent pathways	Qu et al. (2024)
Sini San	Bupleurum chinense DC. [Apiaceae; Bupleuri radix], Paeonia lactiflora Pall. [Paeoniaceae; Paeoniae radix alba], Citrus aurantium L. [Rutaceae; Aurantii immaturus fructus], and Glycyrrhiza uralensis Fisch. [Fabaceae; Glycyrrhizae radix et rhizoma]	HPLC,CCl4-induced mice,network pharmacology,HepG2 cells	Inhibiting AKT-mediated hepatocyte apoptosis	Jiang et al. (2023a)
Danggui Shaoyao San	Angelica sinensis (Oliv.) Diels [Apiaceae; Angelicae sinensis radix], Paeonia lactiflora Pall. [Paeoniaceae; Paeoniae radix alba], Ligusticum chuanxiong Hort. [Apiaceae; Chuanxiong rhizoma], Alisma plantago-aquatica L. [Alismataceae; Alismatis rhizoma], Poria cocos (Schw.) Wolf [Polyporaceae; Poria], and Atractylodes macrocephala Koidz.[Asteraceae; Atractylodis macrocephalae rhizoma]	16S rRNA gene sequencing and untargeted fecal metabolomics,CCl4-induced mice	Modulating gut microbiota	Zhao et al. (2024b)
Hospital-based TCM empirical formulas				
Qijia Rougan formula	Astragalus membranaceus (Fisch.) Bge. [Fabaceae; Astragali radix], Angelica sinensis (Oliv.) Diels [Apiaceae; Angelicae sinensis radix], Amyda orbicularis [Testudinata; Trionycis carapax], Eupolyphaga sinensis Walker [Phthiraptera; Eupolyphagae corporis], Salvia miltiorrhiza Bge. [Lamiaceae; Salviae miltiorrhizae radix et rhizoma], Amygdalus persica (L.) Batsch [Rosaceae; Amygdali persicae semen], Carthamus tinctorius L. [Asteraceae; Carthami flos], Ligusticum wallichii [Apiaceae; Ligustici wallichii rhizoma], Sparganium stoloniferum [Typhaceae; Sparganii rhizoma], and Curcuma zedoaria [Zingiberaceae; Curcumae rhizoma]	Adult Sprague-Dawley (SD) rats,RAW264.7 macrophage cell	Influencing macrophage M2 polarization through the JAK1/STAT6-microRNA-23a feedback loop	Zheng et al. (2023b)
Tianhuang formula	Panax notoginseng (Burk.) F.H. Chen [Araliaceae; Notoginseng Radix et Rhizoma], and Coptis chinensis Franch. [Ranunculaceae; Coptidis Rhizoma]	CCl4-induced and MCD diet-induced liver fibrosis model	Inhibiting CCL2-CCR2 axis and MAPK/NF-κB signaling pathway	Lan et al. (2024)
Yangyinghuoxue decoction	Paeonia lactiflora Pall. [Paeoniaceae; Paeoniae radix alba], Bupleurum chinense DC. [Apiaceae; Bupleuri radix], Citrus reticulata Blanco [Rutaceae; Citri reticulatae pericarpium], Paeonia veitchii Lynch [Paeoniaceae; Paeoniae radix rubra], Ligusticum chuanxiong Hort. [Apiaceae; Chuanxiong rhizoma], Rheum palmatum L. [Polygonaceae; Rhei radix et rhizoma], Ziziphus jujuba Mill. [Rhamnaceae; Jujubae fructus], Salvia miltiorrhiza Bge. [Lamiaceae; Salviae miltiorrhizae radix et rhizoma], Angelica sinensis (Oliv.) Diels [Apiaceae; Angelicae sinensis radix], Scutellaria baicalensis Georgi [Lamiaceae; Scutellariae radix], Glycyrrhiza uralensis Fisch. [Fabaceae; Glycyrrhizae radix et rhizoma], Rehmannia glutinosa (Gaertn.) Libosch. ex DC. [Orobanchaceae; Rehmanniae radix], Eupatorium adenophorum Spreng. [Asteraceae; Eupatorii herba], and Fritillaria thunbergii Miq. [Liliaceae; Fritillariae thunbergii bulbus]	Network pharmacology and molecular docking,CCl4-induced mice	Regulation of PI3K/AKT pathway	Bai et al. (2024b)
Qiwei Tiexie capsule	Iron Chippings (Ferrum magnetisatum) [Magnetitum; Ferri magnetisati], Gypsum Rubrum (Gypsum rubrum) [Sulfatophora; Gypsi rubri], Racemose Inula (Inula racemosa) [Asteraceae; Inulae flos], Costusroot (Saussurea costus) [Asteraceae; Costus radix], Herba Dracocephali Tangutici (Dracocephalum tanguticum) [Lamiaceae; Herbae Dracocephali tangutici], Flos Carthami (Carthamus tinctorius) [Asteraceae; Carthami flos], and Trogopterus Dung (Trogopterus xanthogaster) [Trogopteridae; Trogopteri feces]	CCl4-induced mice,HSC-T6 cells	The suppression of NLRP3 inflammasome activity	Wang et al. (2024b)
Gentiana decoction	Gentianae Scabrae Radix [Gentianaceae; Gentianae Scabrae Radix], Scutellariae Radix [Lamiaceae; Scutellariae Radix], Gardeniae Fructus [Rubiaceae; Gardeniae Fructus], Alismatis Rhizoma [Alismataceae; Alismatis Rhizoma], Mutong [Lardizabalaceae; Mutong], Plantaginis Semen [Plantaginaceae; Plantaginis Semen], Angelicae Sinensis Radix [Apiaceae; Angelicae Sinensis Radix], Bupleuri Radix [Apiaceae; Bupleuri Radix], Rehmanniae Radix [Orobanchaceae; Rehmanniae Radix], and Glycyrrhizae Radix et Rhizoma [Fabaceae; Glycyrrhizae Radix et Rhizoma]	CCl4-induced mice	Upregulating the expression of Parkin	Deng et al. (2024)
Ba-Qi-Rougan formula	Morindae Officinalis Radix[Rubiaceae; Morindae Officinalis Radix], Astragali Radix[Fabaceae; Astragali Radix], Rehmanniae Radix[Orobanchaceae; Rehmanniae Radix], Lycopi Herba[Lamiaceae; Lycopi Herba], Wenyujin Rhizoma Concisum[Zingiberaceae; Wenyujin Rhizoma Concisum], and Citri Reticulatae Pericarpium Viride[Rutaceae; Citri Reticulatae Pericarpium Viride]	UPLC-Q-TOF-MS,CCl4-induced mice,LO2 cells and LX-2 cells	Modulating the MSMP/CCR2/PI3K pathway	Xue et al. (2024)
Wuling capsule	Bupleuri Radix[Apiaceae; Bupleuri radix], Ganoderma lucidum Karst.[Polyporaceae; Ganoderma lucidum], Salvia miltiorrhiza Bunge [Lamiaceae; Salviae miltiorrhizae radix et rhizoma], and Schisandra chinensis Baill.[Schisandraceae; Schisandrae chinensis fructus]	CCl4-induced mice, RAW264.7 cells	Inhibiting of TLR4-NF-κB signaling pathway	Ren et al. (2024)
Ganfule capsules	Codonopsis Radix[Campanulaceae; Codonopsis Radix], Trionycis Carapax[Geoemydidae; Trionycis Carapax], Paridis Rhizoma[Melanthiaceae; Paridis Rhizoma], Atractylodis Macrocephalae Rhizoma [Asteraceae; Atractylodis Macrocephalae Rhizoma], and Astragali Radix[Fabaceae; Astragali Radix]	C57BL/6 mice	Inhibition of glutamine metabolism	Ke et al. (2022)
Yi-Qi-Jian-Pi formula	Stellaria dichotoma L. [Caryophyllaceae; Stellariae radix], Poria cocos (Schw.) Wolf [Polyporaceae; Poria], Scutellaria baicalensis Georgi [Lamiaceae; Scutellariae radix], Citrus reticulata Blanco [Rutaceae; Citri reticulatae pericarpium], Atractylodes macrocephala Koidz. [Asteraceae; Atractylodis macrocephalae rhizoma], Ligustrum lucidum Trew [Oleaceae; Ligustri lucidi fructus], and Angelica sinensis (Oliv.) Diels [Apiaceae; Angelicae sinensis radix]	Network pharmacology, CCl4-induced mice	Inhibiting TGF-β/Smad3 signaling pathway	Yang et al. (2024)
Huangqi Decoction	Astragali Radix[Fabaceae; Astragali Radix], Glycyrrhizae Radix et Rhizoma[Fabaceae; Glycyrrhizae Radix et Rhizoma]	LO2 cells and LX-2 cells	Modulating the lncRNA-C18orf26-1/miR-663a/TGF-β signaling axis	Dong et al. (2023a)
Fufang Muji Granules	Coriolus versicolor extract, Sophora tonkinensis Gagnep. [Fabaceae; Sophorae tonkinensis radix], Cuscuta chinensis Lam. [Convolvulaceae; Cuscutae semen], Juglans mandshurica Maxim. [Juglandaceae; Juglandis cortex]	Untargeted metabolomics ,CCl4-induced mice	Modulating TGF-β1/Smad signaling pathway	Men et al. (2024)
Danhongqing formula	Salvianolic acid B (Sal-B), salidroside (Salid), and artesunate (Art)	Mdr2 mice,cholangiocytes and HSCs	Downregulating long non-coding RNA H19	Li et al. (2024a)

### 6.1 The “guidelines for diagnosis and treatment of hepatic fibrosis with integrated traditional Chinese and Western medicine (2019 edition)”

(Xu and Liu, 2020) highlighted several patented TCM formulas with demonstrated efficacy. In a single-arm clinical trial using objective performance criteria, 251 patients with chronic hepatitis B (CHB) and advanced liver fibrosis underwent 48 weeks of combined therapy with Fuzheng Huayu (FZHY) tablets and entecavir (ETV). The FZHY group showed a significantly higher rate of fibrosis improvement—15% greater than with ETV monotherapy (Zhao et al., 2022). A comprehensive review (Zhou et al., 2024) concluded that FZHY is broadly applicable for treating liver fibrosis across various chronic liver diseases, with numerous animal and cellular studies confirming its effectiveness in targeting all known pathogenic mechanisms of fibrosis. (Dai et al., 2023). reported that Liver Qi Stagnation and Spleen Deficiency Syndrome were the predominant TCM syndromes among patients treated with FZHY. In a study of 400 CHB patients (Liu Y. Q. et al., 2023), compared ETV monotherapy with a combination of ETV and Anluohua Xianwan (ALHX), finding a significantly higher rate of fibrosis regression in the combination group. Similarly, a randomized controlled trial involving 270 CHB patients with early-stage liver fibrosis demonstrated that 48 weeks of Anluo Huaxian Pills (AHP) significantly improved fibrosis compared to placebo, with no notable adverse effects observed in the AHP group (Xiao et al., 2022). In another multicenter, randomized, double-blind, placebo-controlled trial, (Rong et al., 2022), showed that 72 weeks of ETV combined with Biejia-Ruangan (BR) significantly enhanced fibrosis regression in 500 patients with advanced CHB-related liver fibrosis. Preclinical studies support these findings. In rat models, Ganshuang Granules (GSG) reduced key fibrosis biomarkers, and *in vitro* studies indicated that its active compound, Naringin, may attenuate fibrosis by modulating the TGF-β/Smad signaling pathway (Wang F. et al., 2024). Although FZHY has been extensively studied-particularly in patients with Liver Qi Stagnation and Spleen Deficiency-the international persuasiveness of this evidence remains limited. Future research should focus on improving trial design and further exploring TCM syndrome differentiation to strengthen the global acceptance and methodological rigor of related studies. Meanwhile, clinical research on other formulas such as GSG and Biejia-Ruangan remains scarce, with even fewer studies elucidating their mechanisms of action.

### 6.2 Integration of other TCM formulas with biomedicine medicines for liver fibrosis

(Xing et al., 2023) evaluated 240 patients with advanced CHB-related fibrosis and found that 48 weeks of combination therapy with ETV and Ruangan Granule (RG) significantly reduced fibrosis severity. In a retrospective study, (Yu and Ge, 2024), reported that Bushen Huayu Decoction combined with ETV produced greater reductions in fibrosis markers than ETV alone, with statistically significant improvements observed at both 2 and 4 weeks. (Bi et al., 2023), including 22 randomized controlled trials, showed that commercial Chinese polyherbal preparation (CCPP) combined with ursodeoxycholic acid (UDCA) was more effective for primary biliary cholangitis (PBC)-related fibrosis than either treatment alone. In the future, more high-quality clinical studies can be carried out to further verify the efficacy of integrated traditional Chinese and biomedicine medicines, and provide patients with more effective treatment options.

### 6.3 Classic TCM formulas

Mechanistic studies continue to advance understanding of TCM therapies. (He et al., 2024). found that Dahuang Zhechong pills ameliorate liver fibrosis by modulating gut microbiota and associated metabolites. (Huang et al., 2023). demonstrated that Jiawei Taohe Chengqi Decoction inhibits HSC activation *via* the Src/ERK/Smad3 signaling pathway. (Ye et al., 2024). further showed that this decoction mitigates fibrosis by targeting activated HSCs through both the TGF-β1/CUGBP1 and IFN-γ/Smad7 pathways. In addition, (Shao et al., 2024), revealed that the same decoction suppresses HSC activation via inhibition of the Notch signaling pathway and modulation of macrophage polarization. The Guizhifuling Pill exhibits hepatoprotective effects by regulating the Nrf2/HO-1 antioxidant axis and inhibiting the NF-κB inflammatory signaling pathways (Baogui et al., 2022), as well as modulating the PTEN/AKT/mTOR signaling pathway (Yao et al., 2024; Wu et al., 2024) demonstrated that Tao-Hong-Si-Wu-Tang attenuates liver fibrosis by restoring lipid metabolism, inhibiting long-chain acyl-CoA synthetase 4 (ACSL4), and promoting mitophagy. Similarly, (Qu et al., 2024), reported that Si-Wu-Tang reduces ECM deposition and liver fibrosis through the H19-associated miRNA pathway. (Wang S. J. et al., 2023). reported that Xiaochaihu Tang alleviates hepatic fibrosis through leptin and Nrf2 signaling pathways. (Jiang M. et al., 2023). showed that Isorhamnetin, a key metabolite of Sini San, prevents the AKT-mediated suppression of FXR expression. (Zhao Y. et al., 2024). that Danggui Shaoyao San ameliorates hepatic fibrosis by modulating gut microbiota and their metabolites, particularly short-chain fatty acids (SCFAs) and bile acids (BAs). Classic TCM formulas have been extensively studied and applied to a variety of diseases, and their best indications are not limited to liver fibrosis. In the future, clinical studies need to be strengthened to better determine its optimal application in the treatment of liver fibrosis.

### 6.4 Hospital-based TCM empirical formulas

The Qijia Rougan formula exerts anti-fibrotic effects by regulating the JAK1/STAT6/microRNA-23a axis through M2 macrophage polarization (Zheng Y. et al., 2023). The Tianhuang formula reduces liver fibrosis by targeting the CCL2/CCR2 axis and downregulating the MAPK-NF-κB pathway (Lan et al., 2024). Yangyinghuoxue decoction mitigates fibrosis via PI3K/Akt pathway activation (Bai Y. M. et al., 2024). Qiwei Tiexie capsule effectively combats hepatic fibrosis by suppressing NLRP3 inflammasome activation while enhancing hydroxyproline content and glutathione peroxidase activity *in vivo* (Wang S. et al., 2024). Gentiana decoction, as reported by (Deng et al., 2024), inhibits HSC activation through the Parkin signaling pathway. The Danhongqing formula exhibits anti-fibrotic activity by downregulating long non-coding RNA H19, promoting LX-2 cell apoptosis, and inhibiting HSC activation (Li M. et al., 2024; Xue et al., 2024) showed that the Ba-Qi-Rougan formula suppresses activated HSCs via the CCR2/PI3K/AKT signaling pathway. Wuling capsules activate the NF-κB signaling cascade, a critical regulator of inflammation and immunity, thereby reducing hepatic fibrosis (Ren et al., 2024). Ganfule capsules inhibit glutamine metabolism linked to NF-κB signaling, contributing to anti-fibrotic effects (Ke et al., 2022). Fufang Muji granules improve hepatic fibrosis by modulating the TGF-β1/Smad pathway, inhibiting apoptosis, regulating metabolism, and alleviating oxidative stress and inflammation (Men et al., 2024). The Yi-Qi-Jian-Pi formula reverses liver fibrosis by inhibiting the TGF-β/Smad3 axis and modulating the abundance of *Calditerrivibrio nitroreducens*, which negatively correlates with 18β-glycyrrhetinic acid (Yang et al., 2024). Huangqi Decoction exerts anti-fibrotic effects by regulating the lnc-C18orf26-1/miR-663a/TGF-β axis (Dong B. S. et al., 2023).

Currently, the majority of PubMed-indexed studies on TCM formulas are experimental, focusing primarily on elucidating molecular mechanisms. These findings underscore a pressing need for the global research community to clarify the mechanisms of action of TCM formulas through robust scientific inquiry formulas. Future efforts should prioritize rigorous clinical trials to validate these formulas and improve their scientific credibility and global acceptance. Furthermore, formulas derived from botanical drug metabolites may offer enhanced therapeutic efficacy compared to traditional TCM formulas. As such, expanding clinical trials to evaluate both metabolite-based formulas is of substantial significance.

## 7 Discussion

Liver fibrosis, a common manifestation of chronic liver diseases, remains a major clinical challenge due to the absence of targeted anti-fibrotic therapies. The diversity of potential therapeutic agents highlights the urgent need for continued research. This review systematically compiles clinical and experimental studies and demonstrates the efficacy of various treatment medicines.

Previous reviews have primarily concentrated on fundamental research involving individual compounds, such as single drugs, botanical drug metabolites, or potential therapeutic molecules (Zhang A. et al., 2023; Dan et al., 2024; Wenbo et al., 2024), meta-analyses of clinical studies on TCM formulas (Bi et al., 2023; Zhou et al., 2024),as well as. However, relatively few have focused on clinical trials. This review adopts a broader scope, aiming to identify current trends, major research directions, and recent advancements in liver fibrosis. By first offering a comprehensive overview and subsequently exploring targeted areas in depth, this approach is intended to enhance the efficiency of drug development and guide liver fibrosis research with greater precision. Moreover, it provides future researchers with a structured framework to support more informed decision-making in the development of hepatic fibrosis therapies. Nonetheless, this review has several limitations.

1. Broad coverage with limited depth: While this review offers an extensive overview of therapeutic strategies for liver fibrosis—including conventional biomedicine medicines, repurposed agents, potential small molecules, botanical drug metabolites, and TCM formulas, as well as their integration with MSC-ex and NPs—the level of detail is constrained by space. Consequently, although the current status of each category is objectively summarized, the depth of analysis remains limited.
2. Lack of standardized inclusion criteria for TCM formulas: Although clinical and preclinical studies of TCM formulas are thoroughly discussed, the absence of unified criteria for evaluating efficacy limits the reproducibility and comparability of findings. Additionally, many clinical studies fail to investigate underlying mechanisms in detail, while mechanistic studies often lack validation from clinical data, resulting in a disconnect between basic and clinical research.
3. Insufficient elaboration on phenotypes and mechanisms: The review identifies several relevant phenotypes and mechanistic pathways associated with botanical drug metabolites in the treatment of liver fibrosis. However, the limited scope and brevity preclude a comprehensive synthesis. The primary aim is to propose a novel framework for organizing existing findings, offering new insights to aid in the identification of highly effective botanical metabolites.
4. Limited research on combination therapies: The clinical research of the combination of TCM formulas and Biomedicine medicines and the experimental research related to MSC-ex and NPs are relatively abundant, However, clinical trial data for the latter are insufficient. Moreover, the number of combined application articles included in this review is limited. This situation makes it difficult to conduct effective comparative analyses of different combination treatments.

To address these limitations, we propose the following research priorities for future studies.

1. Focused investigation into high-efficacy treatments: Building on the comparative analysis of current therapeutic strategies presented in this review, future studies should concentrate on a specific treatment modality to accelerate clinical translation and improve the adoption of effective therapies for liver fibrosis.
2. Standardization and clinical validation of TCM formulas: There is an urgent need for multicenter, large-scale clinical trials grounded in evidence-based methodologies and supported by bioinformatics tools. These efforts should aim to standardize TCM formulas, establish unified clinical efficacy evaluation systems, optimize prescriptions, and verify both the efficacy and safety of specific formulas, including determination of appropriate dosages.
3. Clinical advancement of botanical drug metabolites: Once specific phenotypes and mechanisms of action are elucidated, clinical trials must be promptly initiated to bridge the gap between basic research and clinical application. Although mechanistic studies in this area are abundant, the lack of corresponding clinical trials hampers comprehensive evaluation and practical advancement of botanical drug metabolites in liver fibrosis therapy.
4. Enrich Clinical Trials of Combination Therapies: To strengthen the evidence base for integrated traditional Chinese and biomedicine medicine treatments, future efforts should prioritize multicenter, randomized controlled trials (RCTs). These trials must be rigorously designed to generate reliable, evidence-based outcomes. Simultaneously, advanced preclinical studies and clinical trial frameworks for MSC-ex and NPs should be refined to optimize their application in combination therapies, thereby enhancing both therapeutic efficacy and safety.

## 8 Conclusion

This review provides a comprehensive analysis of the pathological mechanisms underlying liver fibrosis, systematically sorts out various therapeutic medicines, and analyzes its research status. However, there are some gaps in existing studies, which can easily lead to one-sided research results and affect the comprehensive grasp of the treatment of liver fibrosis. Moreover, there is a critical need to expand the scale and depth of clinical trials, continuously refine liver fibrosis models, and work toward greater standardization and normalization in pharmacological research. These efforts will collectively accelerate the development of effective, targeted treatments for liver fibrosis.
